# Nursing and the Sustainable Development Goals (SDGs): An Essential Commitment

**DOI:** 10.1590/0034-7167.2023760601

**Published:** 2023-12-04

**Authors:** Monica Taminato, Hugo Fernandes, Dulce Aparecida Barbosa

**Affiliations:** I Universidade Federal de São Paulo. São Paulo, São Paulo, Brazil

The Sustainable Development Goals (SDGs) are a global initiative launched by the United Nations (UN) in September 2015 as part of the 2030 Agenda for Sustainable Development, whose objectives summarize a universal call to action to end poverty, protect the environment and ensure that all people have access to opportunities and well-being, all in an equitable and sustainable way^(1-2)^. The 17 interconnected goals and 169 specific targets covering a wide range of social, economic and environmental issues were developed in pursuit of overcoming the most pressing challenges facing the world, including poverty, hunger, health, education, gender equality, clean water, sanitation, affordable energy, economic growth, decent work, reduced inequalities, sustainable cities, climate action, life in water, life on land, peace, justice, among others^(2)^.

Such goals provide a roadmap for governments, civil society organizations, the private sector and individuals to work together to address complex challenges and build a more just, prosperous and sustainable world for present and future generations^(1-2)^. That said, health professionals, as active members of the complex global socio-environmental system, are invited to make every possible effort to achieve the SDGs. Particularly nursing, as it is the health care profession dedicated to care, promotion, prevention, treatment and rehabilitation of people, families and communities, plays a leading role in achieving the SDGs^(3)^.

During the COVID-19 pandemic, we observed a significant number of people without access to interventions and health care. Global inequalities have intensified, catastrophically affecting the most vulnerable groups, placing hundreds of millions of people in extreme poverty^(4)^. The SDGs have been put to the test and, unfortunately, many global risk factors have not changed, leading to permanent alert for future health emergencies and potential new pandemics^(5)^. Therefore, more than ever, the commitment to implementing concrete actions in relation to achieving the SDGs is necessary.

We highlighted, specifically, that nursing contributes significantly to SDG 3, which aims to ensure a healthy life and promote well-being for everyone at all ages. Nurses, technicians, and nursing assistants are on the front lines of health care, providing direct care to patients, performing health screenings, administering medications, and educating people about disease prevention^(3)^. They play a key role in promoting health and preventing illness, helping to achieve goals related to infant mortality, maternal health and disease control. This also includes antimicrobial resistance reduction, which, although not explicit in this SDG, directly impacts well-being, longevity and quality of life in the world, and there must be a high-level commitment from nurses and other health professionals to increase the surveillance capacity and provide quality data as well as actions to ensure its rational and accurate use^(3,6)^.

Nursing is also equally protagonist in SDGs 4 and 5, which seek to ensure quality education for all and achieve gender equality and empower all women and girls^(3)^. Nurses not only care for patients, but also play a vital role in educating patients and their families about health conditions, treatments and preventive measures, thereby contributing to promoting a broader understanding of health. Regarding gender equality, the nursing profession is dominated by women, and many nurses are leaders in their fields. They represent potentially inspiring leadership models for young women, and ultimately promote gender equality in the workplace and in society at large.

Drinking water and basic sanitation promotion (SDG 6) also relies on the active role of nurses, which ranges from educational measures for the potability of water for human consumption^(7)^ to participation in political positions that ensure decision-making that can promote waste treatment. Also noteworthy is the growing role of nurses in health surveillance, which directly contributes to ensuring water quality and reducing risks associated with infection or infestation transmission.

SDG 8 (decent work and economic growth) also stands out, as nursing is one of the professions that contributes significantly to the global economy, creating jobs and opportunities for decent work around the world^(3,6)^. However, it is worth highlighting that the organization and struggle of this professional category is important so that minimum rights are guaranteed, such as minimum wages so that work occurs fairly and without exploitation.

The ethical and moral commitments assumed by this professional category stand out in broad accordance with SDGs 10 and 17, which seek to reduce inequalities within and between countries and the formation of partnerships to improve the health system. These objectives are pursued by nursing on a daily basis, by providing accessible and quality healthcare for everyone, especially the most vulnerable, with interprofessional and intersectoral collaboration, helping to reduce access disparities and intrinsically aligned with the doctrinal principles of the Brazilian Health System (SUS - *Sistema Único de Saúde*) as well as similar models existing around the world^(3,6)^.

Thus, nursing is intrinsically aligned and committed to the SDGs in its daily lives, acting as an essential pillar in promoting health and combating inequalities, playing a vital role in promoting health, gender equality, reducing inequalities and even building more just and peaceful societies, by ensuring equal access to quality health care. Surely, nurses, nursing assistants and technicians are fundamental agents in transforming global goals into tangible actions and promoting a healthier, more equitable and sustainable world.
